# nor-BNI Antagonism of Kappa Opioid Agonist-Induced Reinstatement of Ethanol-Seeking Behavior

**DOI:** 10.1155/2016/1084235

**Published:** 2016-11-06

**Authors:** Erin Harshberger, Emily A. Gilson, Kelli Gillett, Jasmine H. Stone, Laila El Amrani, Glenn R. Valdez

**Affiliations:** Department of Psychology, Grand Valley State University, Allendale, MI, USA

## Abstract

Recent work suggests that the dynorphin (DYN)/kappa opioid receptor (KOR) system may be a key mediator in the behavioral effects of alcohol. The objective of the present study was to examine the ability of the KOR antagonist norbinaltorphimine (nor-BNI) to attenuate relapse to ethanol seeking due to priming injections of the KOR agonist U50,488 at time points consistent with KOR selectivity. Male Wistar rats were trained to self-administer a 10% ethanol solution, and then responding was extinguished. Following extinction, rats were injected with U50,488 (0.1–10 mg/kg, i.p.) or saline and were tested for the reinstatement of ethanol seeking. Next, the ability of the nonselective opioid receptor antagonist naltrexone (0 or 3.0 mg/kg, s.c.) and nor-BNI (0 or 20.0 mg/kg, i.p.) to block U50,488-induced reinstatement was examined. Priming injections U50,488 reinstated responding on the previously ethanol-associated lever. Pretreatment with naltrexone reduced the reinstatement of ethanol-seeking behavior. nor-BNI also attenuated KOR agonist-induced reinstatement, but to a lesser extent than naltrexone, when injected 24 hours prior to injections of U50,488, a time point that is consistent with KOR selectivity. While these results suggest that activation of KORs is a key mechanism in the regulation of ethanol-seeking behavior, U50,488-induced reinstatement may not be fully selective for KORs.

## 1. Introduction

High rates of relapse following periods of abstinence are typically experienced by many alcoholics, contributing to the challenge of the long-term management of alcoholism. The underlying neuropharmacological mechanisms associated with relapse, however, are not yet fully understood. Recent work suggests that the dynorphin (DYN)/kappa opioid receptor (KOR) system may be a key mediator in the behavioral effects of alcohol and other drugs of abuse [1] including relapse.

Previous work has shown that KOR antagonists block stress induced reinstatement of cocaine seeking and nicotine seeking in rats [2, 3]. The selective KOR antagonist norbinaltorphimine (nor-BNI) also reverses reinstatement of heroin seeking by the pharmacological stressor yohimbine [4]. Additionally, activation of KORs via KOR agonists reinstates cocaine-seeking behavior in squirrel monkeys via corticotropin-releasing factor (CRF) and noradrenergic mechanisms that are commonly associated with stress [5]. Taken together, these results suggest that KORs may play a significant role in the stress-related effects of abstinence.

KORs may also regulate the reinstatement of ethanol-seeking behavior. For example, the KOR antagonist JDTic reduces cue-induced ethanol reinstatement when administered 2 hours prior to testing [6, 7]. nor-BNI has also been shown to reduce ethanol seeking in rats when administered 2 hours, but not 24 hours, prior to treatment with the KOR agonist U50,488 [8]. Although these data demonstrate that KOR antagonists reduce the reinstatement of ethanol-seeking behavior, this effect appears to occur at time points when these antagonists do not selectively bind to KORs. Previous work has shown that KOR antagonists are most selective for KORs at 24 hours after administration and are nonselective opioid receptor antagonists at earlier times [9–11]. Because these effects of KOR antagonists were found when administered less than 24 hours after injection, it is unclear if this effect is specifically due to their blockade of KORs or to nonselective opioid receptor antagonism. The objective of the present study was to further examine the mechanisms by which priming injections of the KOR agonist U50,488 reinstate ethanol seeking. Nonselective opioid receptor mechanisms will be explored by investigating the ability of naltrexone, an antagonist with binding affinity for mu opioid receptors (MORs), delta opioid receptors (DORs), and KORs [12], to block U50,488-induced reinstatement of ethanol-seeking behavior. In order to determine the specific role of KORs in U50,488-induced reinstatement, the ability of nor-BNI to reduce ethanol seeking at time points that are consistent with KOR selectivity will also be examined.

## 2. Materials and Methods

### 2.1. Animals

Male Wistar rats (Charles River, Portage, MI; *n* = 26) were used in this experiment. Body weights were 200–250 g and age was approximately 60 days at the start of the experiments. Rats were group housed (2-3 per cage) with food and water available* ad libitum* except during the initial 3 days of ethanol self-administration training and were weighed daily. Rats were maintained on a 12-hour light/dark cycle (lights on at 22:00 h). Procedures met guidelines of the National Institutes of Health* Guide for the Care and Use of Laboratory Animals* (NIH Publication number 85-23, revised 2011) and were approved by the Institutional Animal Care and Use Committee of Grand Valley State University.

### 2.2. Drugs and Injections

U50,488 ((*trans*)-3,4-dichloro-*N*-methyl-*N*-[2-(1-pyrrolidinyl)-cyclo-hexyl] benzeneacetamide; Tocris Biosciences, Ellisville, MO) and norbinaltorphimine (nor-BNI; Tocris Biosciences, Ellisville, MO) were dissolved in 0.9% saline solution for intraperitoneal (i.p.) injections. Naltrexone (Tocris Biosciences, Ellisville, MO) was dissolved in 0.9% saline solution for subcutaneous (s.c.) injections.

### 2.3. Ethanol Self-Administration Training

Rats were trained to lever press for ethanol using an adapted sweetened solution fading procedure [13], using saccharin that culminates in rats consuming sufficient unsweetened 10% ethanol to produce pharmacologically relevant blood alcohol levels. Standard operant chambers (Med Associates, St. Albans, VT) housed in sound attenuated and ventilated cubicles were used. Syringe pumps (Med Associates, St. Albans, VT) dispensed ethanol into a stainless steel drinking cup mounted 4 cm above the grid floor in the middle of one side panel. Two levers were located 4.5 cm to either side of the drinking cup. Fluid delivery and recording of operant responding were controlled by a computer. A fixed ratio 1 (FR1) schedule of reinforcement was used, with each response on the active lever resulting in the delivery of 0.1 mL of fluid.

At the onset of training, rats were limited to 2 h of access to water in their home cages for 3 days only and were allowed access to the operant boxes where responding on one lever resulted in the delivery of a 0.2% saccharin solution. Similar levels of water restriction have been previously shown to have no effect on physiological and behavioral measures of stress [14]. Thereafter, water restriction was discontinued. Across sessions, ethanol concentrations gradually increased from 5% to 10%, with each concentration first presented with saccharin and then presented alone. During this procedure one lever was active, producing an ethanol/saccharin solution. Responses on the inactive lever were recorded as a measure of general motor activity but had no programmed consequence. Half of the animals received an ethanol/saccharin solution following responses on the left lever and the other half received the solution after a response on the right lever. Daily training sessions were 30 min in duration (Table 1).

### 2.4. Extinction

Responding for ethanol was extinguished by eliminating the delivery of the ethanol solution following responding on the ethanol-associated lever. Extinction sessions were otherwise identical to the self-administration sessions. Extinction sessions were conducted daily until responding declined to ≤10% of the number of responses maintained by ethanol self-administration for at least 3 consecutive sessions.

### 2.5. U50,488-Induced Reinstatement of Ethanol Seeking

**Figure 1:**
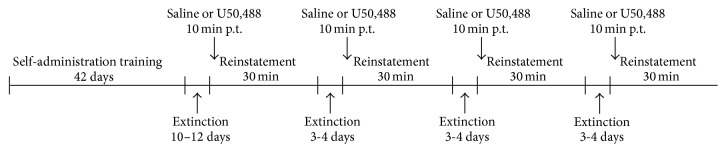
Experimental timeline for U50,488-induced reinstatement testing. Rats (*n* = 13) were trained to self-administer ethanol, and then lever pressing was extinguished. Following extinction rats were injected with U50,488 (0.1–10 mg/kg, i.p.) or saline 10 minutes prior to testing. Tests were conducted every 3-4 days, with rats experiencing extinction sessions daily between test days.

Following extinction, rats (*n* = 13) were injected with priming doses of U50,488 (0.1–10 mg/kg, i.p.) or saline 10 min prior to testing for the reinstatement of ethanol seeking. Rats were then placed in the operant chambers and reinstatement testing occurred under extinction conditions. Tests were conducted every 3-4 days, with rats experiencing extinction sessions daily between test days. Doses were administered in a counterbalanced order using a Latin Square design (Figure 1).

### 2.6. Naltrexone and nor-BNI Antagonism of U50,488-Induced Reinstatement

**Figure 2:**
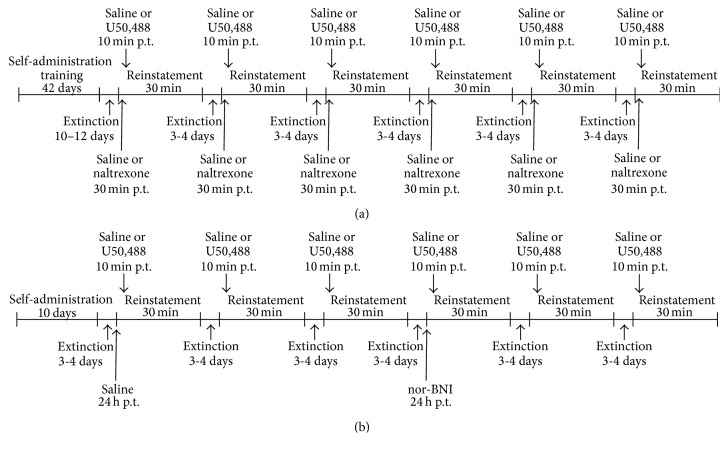
(a) Experimental timeline for naltrexone antagonism of U50,488-induced reinstatement. Rats (*n* = 13) were trained to self-administer ethanol, and then lever pressing was extinguished. Following extinction, rats were pretreated with naltrexone (3.0 mg/kg, s.c.) or saline 30 min prior to testing. Rats were then injected with U50,488 (0.1–1.0 mg/kg, i.p.) or saline 10 min prior to testing. Test sessions were conducted every 3-4 days, with rats experiencing extinction sessions daily between test days. (b) Experimental timeline for nor-BNI antagonism of U50,488-induced reinstatement. Stable self-administration levels were reestablished following the experiment described in (a) and then extinguished. Following extinction, rats were pretreated with a single saline injection. 24 h later rats were injected with a U50,488 (0.1–1.0 mg/kg, i.p.) or saline 10 minutes prior to testing. Test sessions were repeated every 3-4 days with rats experiencing extinction sessions daily between test days. After all doses of U50,488 were tested, rats were given a single injection of nor-BNI (20 mg/kg, i.p.). 24 h later, rats were injected with U50,488 (0.1–1.0 mg/kg, i.p.) or saline 10 minutes prior to testing. Test sessions were repeated every 3-4 days with rats experiencing extinction sessions daily between test days.

A separate group of rats (*n* = 13) were trained to self-administer ethanol and then underwent extinction as described. Following extinction, rats were pretreated with naltrexone (3.0 mg/kg, s.c.) or saline 30 min prior to reinstatement testing. Rats were then injected with priming doses of U50,488 (0.1–1.0 mg/kg, i.p.) or saline 10 min prior to testing. Test sessions were conducted every 3-4 days, with rats experiencing extinction sessions daily between test days. The dose range of U50,488 was adjusted based on the results of the previous experiment, and doses were administered in a counterbalanced order using a Latin Square design (Figure 2(a)).

After all dose combinations were tested in each rat, stable self-administration levels were reestablished for 2 weeks and then once again extinguished. One rat was excluded from this phase of the experiment for failing to reestablish stable levels of ethanol self-administration. Following extinction, rats were pretreated with a single saline injection. The first reinstatement test session occurred 24 h later when rats were injected with a priming dose of U50,488 (0.1–1.0 mg/kg, i.p.) or saline 10 min prior to testing. Over the next 6–8 days, test sessions were repeated every 3-4 days with rats experiencing extinction sessions daily between test days. Doses of U50,488 were administered in a counterbalanced order using a Latin Square design. No additional saline injections were given over this testing period. After all doses of U50,488 were tested, rats were given a single injection of nor-BNI (20 mg/kg, i.p.). 24 h later, rats were injected with a priming dose of U50,488 (0.1–1.0 mg/kg, i.p.) or saline 10 min prior to testing. A pretreatment time of 24 h was chosen because previous research has shown that nor-BNI is most selective for KORs at 24 h after administration as opposed to earlier times [9]. Over the next 6–8 days, test sessions were repeated as described. No additional injections of nor-BNI were administered during this time period because the selective KOR antagonist effects of nor-BNI have been shown to last for up to 30 days after initial administration [9, 11, 15] (Figure 2(b)). Saline and nor-BNI pretreatment occurred in this order because nor-BNI has been shown to be an irreversible antagonist [15].

### 2.7. Data Analysis

Data for the experiment examining the reinstatement of ethanol seeking by U50,488 was analyzed using a one-way repeated measures analysis of variance (ANOVA) with U50,488 dose as the within subjects factor. Post hoc analysis was performed using Tukey's test to determine any significant differences between treatment conditions. Data for the antagonism experiments were analyzed using a two-way repeated measures ANOVA with naltrexone or nor-BNI dose and U50,488 dose as the within subjects factors. Sidak's test for multiple comparisons was used for post hoc analysis to determine if there were any significant differences between saline and naltrexone or saline and nor-BNI when rats were injected with U50,488. The alpha level for all statistical analysis was *p* < 0.05.

## 3. Results

**Figure 3:**
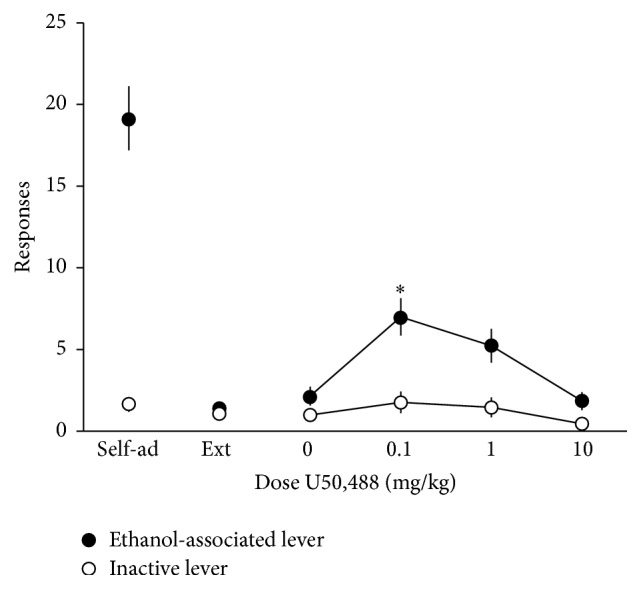
U50,488 reinstates ethanol-seeking behavior. Rats (*n* = 13) were trained to self-administer a 10% ethanol solution using standard operant procedures, and then lever pressing for ethanol was subsequently extinguished. Responding on an inactive lever was measured as an indication of general motor activity. Following extinction, rats received priming injections of U50,488 (0–10 mg/kg, i.p.) and were tested for reinstatement of responding on the previously ethanol-associated lever. Tests were conducted every 3-4 days, with rats experiencing extinction sessions daily between test days. Data are expressed as the mean number of lever responses ± SEM during ethanol self-administration sessions. ^*∗*^ *p* < 0.05 compared to self-administration, extinction, and 10.0 mg/kg U-50,488 groups, Tukey's test.

Injections of U50,488 significantly increased responding on the previously ethanol-associated lever (*F*(3,28) = 7.94, *p* < 0.05, Figure 3) without affecting responding on the inactive lever. Further analysis revealed that rats receiving the 0.1 mg/kg dose of U50,488 responded more on the previously ethanol-associated lever compared to rats injected with saline or 10 mg/kg U50,488 (*p* < 0.05, Tukey's test).

**Figure 4:**
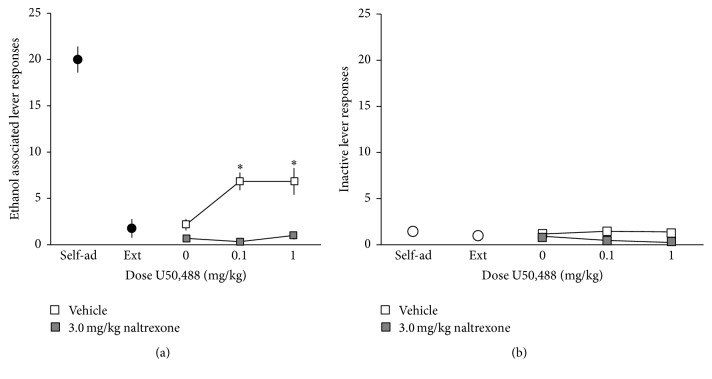
(a) Naltrexone blocks U50,488-induced reinstatement of ethanol seeking. Rats (*n* = 13) were trained to self-administer a 10% ethanol solution using standard operant procedures, and then lever pressing for ethanol was subsequently extinguished. Following extinction, rats were pretreated with naltrexone (0 or 3.0 mg/kg, s.c.) 30 min prior to reinstatement testing. Prior to testing, rats received priming injections of U50,488 (0–1.0 mg/kg, i.p.) and were tested for reinstatement of responding on the previously ethanol-associated lever. Tests were conducted every 3-4 days, with rats experiencing extinction sessions daily between test days. Data are expressed as the mean number of lever responses ± SEM during ethanol self-administration sessions. ^*∗*^ *p* < 0.05 compared to saline-injected rats receiving the same dose of U50,488, Sidak's test for multiple comparisons. (b) Responding on an inactive lever was measured as an indication of general motor activity. No effects on inactive lever responding were observed.

There were significant main effects of U50,488 dose (*F*(2,24) = 5.38, *p* < 0.05) and naltrexone dose (*F*(1,12) = 39.21, *p* < 0.0001) on responses on the previously ethanol-associated lever, as well as a significant interaction between U50,488 and naltrexone on ethanol-associated lever responding (*F*(2,24) = 7.56, *p* < 0.01, Figure 4(a)). Post hoc analysis revealed that pretreatment with naltrexone significantly decreased U50,488-induced responding on the ethanol-associated lever (*p* < 0.05, Sidak's test for multiple comparisons). There were no effects on responding on the inactive lever (Figure 4(b)).

**Figure 5:**
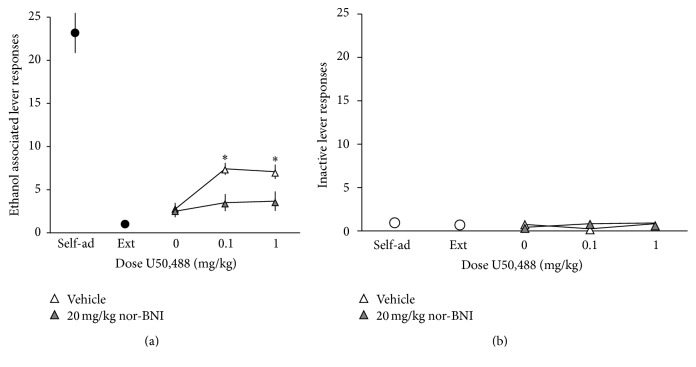
(a) nor-BNI attenuates U50,488-induced reinstatement of ethanol seeking. Rats (*n* = 12) were trained to self-administer a 10% ethanol solution using standard operant procedures, and then lever pressing for ethanol was subsequently extinguished. Following extinction, rats were pretreated with saline 24 h prior to reinstatement testing. Prior to testing, rats received priming injections of U50,488 (0–1.0 mg/kg, i.p.) and were tested for reinstatement of responding on the previously ethanol-associated lever. Over the next 6–8 days, test sessions were repeated every 3-4 days with rats experiencing extinction sessions daily between test days, and doses of U50,488 were given in an irregular order. After all doses of U50,488 were tested, rats were injected with the KOR antagonist nor-BNI (20 mg/kg, i.p.). 24 h later, rats were injected with a priming dose of U50,488 (0.1–1.0 mg/kg, i.p.) or saline prior to testing. Over the next 6–8 days, test sessions were repeated as described. Data are expressed as the mean number of lever responses ± SEM during ethanol self-administration sessions. ^*∗*^ *p* < 0.05 compared to saline-injected rats receiving the same dose of U50,488, Sidak's test for multiple comparisons. (b) Responding on an inactive lever was measured as an indication of general motor activity. No effects on inactive lever responding were observed.

There were also significant main effects of U50,488 dose (*F*(2,22) = 7.75, *p* < 0.01) and nor-BNI dose (*F*(1,11) = 13.73, *p* < 0.01) on ethanol-associated lever responding. Although not statistically significant, there was a trend towards an interaction between nor-BNI and U50,488 on lever responses for the previously ethanol-associated lever (*F*(2,22) = 2.44, *p* = 0.10, Figure 5(a)). Post hoc analysis demonstrated that pretreatment with nor-BNI led to a significant decrease in U50,488-induced responding on the ethanol-associated lever (*p* < 0.05, Sidak's test for multiple comparisons). Inactive lever responses were not affected by any treatment (Figure 5(b)).

## 4. Discussion

The present experiments found that U50,488 reinstates ethanol seeking in rats previously trained to self-administer ethanol. When considered with previous research regarding the role of the KOR/DYN system in enhanced ethanol self-administration [16–18] and cue-induced reinstatement [6, 7], these results further support the hypothesis that direct activation of KORs plays a significant role in ethanol reinstatement. Furthermore, nor-BNI attenuated the reinstatement of ethanol seeking following priming injections of U50,488 at time points consistent with KOR selectivity. However, the magnitude of this effect was lesser than that found when rats were pretreated with naltrexone, suggesting that other opioid receptors may also facilitate the effects of U50,488 on ethanol seeking. While these findings suggest that KORs are involved in mediating KOR agonist-induced reinstatement, this effect may not be selective to KOR activation.

The current experiments found that the KOR antagonist nor-BNI reverses U50,488 induced reinstatement of responding on the previously ethanol-associated lever at time points consistent with selective KOR antagonism. Although this finding parallels recent work demonstrating that KOR antagonists have the ability to attenuate the reinstatement of ethanol seeking, this effect was previously found at time points that may not be consistent with KOR selectivity [6–8]. For example, previous work has found that 10 mg/kg nor-BNI attenuated U50,488-induced reinstatement of ethanol seeking when administered 2 hours prior to reinstatement testing, but not when given 24 hours prior [8]. However, nor-BNI does not appear to act as a selective KOR antagonist until 24 hours after administration and is a nonselective opioid receptor antagonist at earlier times [9–11]. In contrast, the present study found that a single injection of 20 mg/kg nor-BNI attenuated ethanol seeking when administered 24 hours prior to reinstatement testing and that this effect lasted for up to 8 days. This time course is also consistent with the long-lasting ability of KOR antagonists to block the effects of KOR agonists, but not the effects of nonselective opioid agonists [9–11, 15]. One explanation for this discrepancy is the difference in doses of nor-BNI administered. The findings of Funk et al. [8] are consistent with a previous study which found that injections of 10 mg/kg nor-BNI did not reverse KOR agonist-induced reinstatement of cocaine seeking in squirrel monkeys [5]. However, previous data from our laboratory has shown that U50,488 induced anxiety-like behavior is reduced in the elevated plus maze when rats are injected with 20 mg/kg nor-BNI, but not 10 mg/kg nor-BNI, 24 hours prior to testing [19]. It appears that higher doses of nor-BNI may be required to block the behavioral effects of U50,488 at time points more consistent with KOR selectivity.

Previous work has shown that KOR agonist-induced reinstatement of cocaine-seeking behavior in squirrel monkeys is reversed by naltrexone [5], and the results of the present study extend this finding to ethanol seeking. The ability of naltrexone to prevent reinstatement following injections of U50,488 demonstrates that MORs and DORs may also play a significant role in continued drug seeking following administration of KOR agonists. This result is consistent with previous findings demonstrating that naltrexone attenuates ethanol priming-induced [20] and cue-induced reinstatement [21, 22]. It should also be noted that naltrexone reduced ethanol seeking to a greater extent compared to nor-BNI. Previous work has shown that naltrexone may be more effective than nor-BNI in blocking the KOR agonist-induced drug seeking [5], motor suppression [23], and antinociception [24]. When considered with these previous results, the findings of the current study suggest that although KORs play a key role in regulating KOR-agonist-induced reinstatement, persistent ethanol-seeking behavior following U50,488 administration may not be selectively mediated by KORs.

One hypothesis is that the KOR/DYN system may work in conjunction with other neurotransmitter systems to regulate relapse. For example, type 1 corticotropin-releasing factor (CRF_1_) receptor antagonist CP 154,526 and the *α*_2_ adrenoceptor agonist clonidine significantly attenuate KOR agonist priming-induced reinstatement of drug seeking [5], indicating that this finding is likely due to an interaction between the KOR/DYN system and CRF and noradrenergic systems. Further supporting this hypothesis, U50,488-induced reinstatement of ethanol seeking is attenuated by the CRF_1_ receptor antagonist antalarmin, and nor-BNI reduces reinstatement via priming injection of yohimbine, an *α*_2_ adrenoceptor antagonist [8]. DYN and CRF appear to interact in various regions of the brain [25–27] and stimulate the hypothalamic-pituitary-adrenal axis in rodents [28] and humans [29], which is primarily regulated by CRF release in the hypothalamus [30]. With regard to noradrenergic interactions, kappa opioids have been shown to enhance norepinephrine turnover in the paraventricular nucleus of the hypothalamus [28], increase norepinephrine metabolism in the brain stem and cortex [31], and stimulate norepinephrine release in the hippocampus [32]. Taken together with these interactions observed between the DYN/KOR system and other stress-related neurotransmitter systems, the present study suggests that U50,488 may also work in conjunction with other stress-related systems to reinstate ethanol seeking.

Although U50,488 led to a significant increase in responding on the previously ethanol-associated lever, response rates during reinstatement testing were less pronounced when compared to those observed during ethanol self-administration training. However, this lower rate of responding is consistent with previous work examining KOR agonist-induced reinstatement of drug seeking [5] and may be due, in part, to the motor-suppressive effects of KOR agonists. In the current study, only the 0.1 and 1.0 mg/kg doses of U50,488 led to an increase in responding on the ethanol-associated lever. Rats injected with the 10 mg/kg dose of U50,488 showed response levels similar to those seen during extinction and in rats injected with saline, suggesting that this highest dose may have led to general motor suppression. Previous research has found that U50,488 decreases the number of total arm entries, which is thought to be an index of locomotor activity [33], in rats examined in the elevated plus maze [19, 34]. U50,488 has also been shown to decrease operant response rates [35]. In the current study, U50,488 injections led to an increase in responding on the previously ethanol-associated lever despite these motor-suppressive effects, suggesting that stimulation of KORs plays a significant role in the reinstatement of ethanol seeking.

The current study demonstrated that the KOR agonist, U50,488, increased responding on the previously ethanol-associated lever following extinction, an effect attenuated by nor-BNI at time points consistent with KOR selectivity. These results suggest that KORs are a key mechanism in the reinstatement of ethanol seeking. However, this effect occurred to a lesser degree when compared to naltrexone, indicating that U50,488 may also interact with other neurochemical systems to reinstate ethanol-seeking behavior. When considered with previous findings demonstrating that KOR agonists interact with stress-related systems to reinstate drug seeking [5, 8], it appears that U50,488 may act as a pharmacological stressor to induce persistent ethanol seeking.

## Figures and Tables

**Table 1:** Ethanol self-administration training procedure.

Time	Procedure
3 days	0.2% saccharin; 1 lever (limited home cage water access)
2 days	0.2% saccharin; 1 lever
2 days	2% ethanol + 0.2% saccharin; 1 lever
8 days	5% ethanol + 0.2% saccharin versus inactive; 2-lever choice
4 days	5% ethanol versus inactive; 2-lever choice
2 days	8% ethanol + 0.2% saccharin versus inactive; 2-lever choice
2 days	2% ethanol versus inactive; 2-lever choice
4 days	10% ethanol + 0.2% saccharin versus inactive; 2-lever choice
16 days	10% ethanol versus inactive; 2-lever choice
